# Beyond Barriers: Achieving True Equity in Cancer Care

**DOI:** 10.3390/curroncol32060349

**Published:** 2025-06-12

**Authors:** Zaphrirah S. Chin, Arshia Ghodrati, Milind Foulger, Lusine Demirkhanyan, Christopher S. Gondi

**Affiliations:** 1 Department of Internal Medicine, University of Illinois College of Medicine Peoria, Peoria, IL 61605, USA; zchin3@uic.edu (Z.S.C.); aghodr2@uic.edu (A.G.); lusinhd@uic.edu (L.D.); 2 OSF Saint Francis Medical Center, 530 NE Glen Oak Avenue, Peoria, IL 61637, USA; mfoulger24@osfhealthcare.org; 3 Department of Surgery, University of Illinois College of Medicine Peoria, Peoria, IL 61605, USA; 4 Departments of Health Science Education and Pathology, University of Illinois College of Medicine Peoria, Peoria, IL 61605, USA; 5 Health Care Engineering Systems Center, The Grainger College of Engineering, University of Illinois at Urbana-Champaign Urbana Illinois, Urbana, IL 61801, USA

**Keywords:** healthcare disparities, cancer care, racial minorities, clinical trials, health equity

## Abstract

Healthcare disparities in cancer care remain pervasive, driven by intersecting socioeconomic, racial, and insurance-related inequities. These disparities manifest in various forms such as limited access to medical resources, underrepresentation in clinical trials, and worse cancer outcomes for marginalized groups, including low-income individuals, racial minorities, and those with inadequate insurance coverage, who face significant barriers in accessing comprehensive cancer care. This manuscript explores the multifaceted nature of these disparities, examining the roles of socioeconomic status, race, ethnicity, and insurance status in influencing cancer care access and outcomes. Historical and contemporary data highlight that minority racial status correlates with reduced clinical trial participation and increased cancer-related mortality. Barriers such as insurance coverage, health literacy, and language further hinder access to cancer treatments. Addressing these disparities requires a systemic approach that includes regulatory reforms, policy changes, educational initiatives, and innovative trial and treatment designs. This manuscript emphasizes the need for comprehensive interventions targeting biomedicine, socio-demographics, and social characteristics to mitigate these inequities. By understanding the underlying causes and implementing targeted strategies, we can work towards a more equitable healthcare system. This involves improving access to high-quality care, increasing participation in research, and addressing social determinants of health. This manuscript concludes with policy recommendations and future directions to achieve health equity in cancer care, ensuring optimal outcomes for all patients.

## 1. Introduction to Disparities in Cancer Care

Healthcare disparities, characterized by inequitable access to medical resources and health outcomes, remain pervasive in oncology [1]. These disparities are rooted in complex social, economic, and political factors and manifest in various forms, including socioeconomic status, race, ethnicity, and insurance status [2,3]. Cancer care is especially susceptible to these inequities, as it often mirrors broader societal disparities in health.

Historical and contemporary data consistently demonstrate that cancer diagnoses disproportionately impact marginalized populations, including low-income individuals with inadequate insurance coverage and racial minorities [4,5]. While the interplay of these factors makes it difficult to isolate a single primary driver, research has unequivocally established that minority racial status correlates with reduced participation in clinical trials and heightened cancer-related mortality [6,7,8]. Additionally, structural barriers related to insurance coverage, health literacy, and language significantly hinder access to and the utilization of cancer treatments [9,10,11].

In public health discourse, the terms “disparity” and “inequity” are often used interchangeably, yet they carry distinct meanings. A health disparity refers to a measurable difference in health outcomes or access to healthcare services between different population groups. These differences may be observed across various dimensions, such as race, ethnicity, socioeconomic status, gender, or geographic location. While all health inequities are disparities, not all disparities are inequities [8,12,13,14,15,16]. Health inequity is a health disparity that is considered avoidable, unfair, and unjust, stemming from systemic and modifiable social, economic, or environmental disadvantages [17,18]. Thus, while a disparity simply states a factual difference, an inequity highlights the ethical and moral dimension, implying that the observed difference is a result of injustice that could, and should, be remedied through targeted interventions and policy changes. Addressing health inequity is beyond the scope of this article.

The motivation for this review stems from the urgent need to bridge these gaps to achieve true equity in cancer care. A holistic understanding of how intersecting socioeconomic and systemic factors drive these inequities and how to remedy them remains a crucial knowledge gap [19]. This study includes a critical analysis of the roles of patients, providers, healthcare systems, and their interactions [20,21]. By comprehensively considering the extent of disparities across socio-demographic characteristics, we can identify strategic intervention points and prioritize targeted strategies [22].

Interventions to mitigate, reverse, or eliminate these disparities require a multifaceted approach. This may include regulatory reforms, policy changes, educational initiatives, and innovative approaches to trial and treatment design [20,23]. To achieve meaningful change, interventions must target clearly identified causal factors and modifiable variables that contribute to disparities. Current evidence supports targeting disparities related to biomedicine, socio-demographics (e.g., education, residence, insurance status, employment, and life stressors), and certain social characteristics [24,25,26]. While research on other social factors is extensive, their direct relevance to cancer care intervention may be less established [19].

The objective of this review is to explore and synthesize multifaceted drivers of cancer disparities and to highlight practical strategies and policy directions that can move us beyond barriers towards the goal of true equity in cancer care. Though this is not a systematic review, we used the criteria as described in Table 1 to screen the literature during the compilation phase of this research [2,27].

Focused Literature Review on Cancer Disparities: Table 1 presents a focused literature review categorizing key factors contributing to cancer disparities. The included literature was primarily published between January 2000 and December 2023. While most references fall within this timeframe, a limited number of highly impactful and foundational studies published outside this range were also selected to ensure comprehensive coverage and historical context. Each category provides a description of the disparity and its underlying rationale, supported by relevant examples and statistics from the cited literature. The final column indicates the approximate number of references that informed each specific category, reflecting the depth of research considered.

### 1.1. Defining Socioeconomic Disparities

The influence of socioeconomic status (SES) [5,12,16,28,29,30,31,32,33,34,35,36], typically encompassing income, education, and occupation, on an individual’s or family’s access to healthcare, engagement with services, and overall health outcomes is multifaceted and difficult to quantify [36]. SES embodies a range of lived experiences, opportunities, resources, constraints, and social networks that influence health across multiple dimensions, including diet, exercise, housing, stress management, access to preventive care, and health education [25,27,37,38].

Direct financial barriers, such as the inability to afford health insurance or necessary medical treatments, have clear, adverse effects on health [39]. Indeed, the poorest communities within every racial or ethnic population subset have worse outcomes, confirming that poverty is an adverse prognostic factor regardless of racial or ethnic identity [40]. Additionally, evidence suggests that individuals with lower or less prestigious educational attainment or lower income may disproportionately engage in risky health behaviors due to limited access to preventive resources or education [41]. Conversely, those with higher incomes typically benefit from superior healthcare access, including advanced diagnostic services, timely interventions, and increased opportunities for participation in cancer clinical trials. These factors contribute to a documented socioeconomic effect on cancer outcomes, where patients from lower socioeconomic backgrounds are more likely to be diagnosed at later stages, receive less aggressive treatment, and face poorer prognoses regardless of the cancer stage [42].

Recent research by Alderwick and Gottlieb [43] examined the mechanisms underlying the relationship between socioeconomic status (SES) and health outcomes. Despite this progress, the definitions and pathways by which SES influences health remain less clearly articulated [43]. Socioeconomic disparities reflect a complex, interwoven set of factors that are difficult to isolate [44]. Community-level interventions and policy changes are frequently advocated as solutions, though understanding precisely how these interventions reduce disparities requires further clarification.

Evidence also points to factors such as duration of residence, migration patterns, and social capital as being tightly linked to neighborhood environments, further complicating SES analyses [45]. Thus, careful interpretation is required to avoid generalizing the findings from isolated analyses to broader healthcare practices.

### 1.2. Racial and Ethnic Disparities in Cancer Care

Disparities in health outcomes for racial and ethnic minorities are particularly pronounced when it comes to cancer [35]. Black men, for instance, face the highest overall cancer incidence and mortality rates, including elevated rates of prostate, lung, and colorectal cancers compared to other racial groups [46]. Similarly, Native Hawaiian and Pacific Islander populations experience higher incidence and worse outcomes than white individuals, partly because they are diagnosed at later stages [47]. Asian populations, while having lower overall cancer rates, face significant disparities with certain cancers like liver and stomach cancer being more prevalent [48].

Several factors contribute to these disparities. Social determinants of health, including economic stability, education, and neighborhood conditions, substantially influence cancer risks and outcomes [49]. Lower income levels and higher rates of poverty among racial and ethnic minorities limit access to high-quality healthcare and preventive services [50]. Lower educational attainment affects health literacy, making it harder to navigate the healthcare system and understand medical information [51]. Additionally, living in areas with fewer healthcare facilities, higher pollution levels, and limited access to healthy foods increases cancer risk and reduces survival rates [52].

Healthcare access and quality are also significant concerns. Racial and ethnic minorities are more likely to be uninsured or underinsured, leading to delayed diagnoses and treatment [53]. People in these groups often live in areas with fewer healthcare providers and facilities, limiting access to timely and effective cancer care [54]. Moreover, implicit biases among healthcare providers can affect patient interactions and contribute to differences in the quality of care delivered [55].

Additionally, while racial and ethnic disparities in incidence and outcome reflect several factors such as social and economic conditions, disparity trends hold constant when these known risk factors are accounted for, indicating that they do not fully explain the differences between groups [56]. Indeed, African American identity is independently associated with outcome in socioeconomic status-adjusted breast cancer survival, and equal treatments via adjuvant therapy trials result in equal outcomes except in hormonally driven diseases where African American patients had persistent survival disadvantages [28,29]. Thus, a growing body of evidence suggests the urgent need for inclusivity in clinical and biomedical research to better understand ancestral influences, environmental exposures, and social factors [26].

Addressing these disparities requires a multifaceted approach. Efforts to recruit more people of color into cancer research studies are crucial to ensure that the findings are applicable to all populations and to identify specific needs and effective treatments for minority groups [57]. Ensuring equitable access to clinical trials involves addressing logistical barriers, such as transportation and childcare, and providing information in multiple languages [58].

Cultural competence is another important aspect. Training healthcare providers in cultural competence can improve patient–provider interactions and reduce biases [59]. This includes understanding cultural beliefs and practices related to health and illness [60]. Providing culturally tailored education materials can help patients understand their diagnosis and treatment options, leading to better health outcomes [61].

Policy interventions are also essential. Implementing policies that address social determinants of health, such as improving access to education, housing, and employment opportunities, can reduce health disparities [62]. Healthcare system reforms aimed at increasing insurance coverage, expanding access to preventive services, and improving the quality of care in underserved areas are critical [63,64].

Ultimately, addressing cancer disparities among racial and ethnic minorities requires improving access to high-quality care, increasing participation in research, and addressing social determinants of health (Table 2). By recognizing and accounting for the historical and social contexts that contribute to these disparities, effective interventions can be developed to achieve health equity in cancer care [65,66,67].

### 1.3. Insurance-Based Disparities

Access to high-quality cancer care is profoundly influenced by a patient’s insurance status and the specific coverage they hold [71]. Population-level data reveal that individuals with public insurance are at a higher risk of delayed diagnosis, less aggressive therapy, decreased treatment-related surveillance, elevated dropout rates from cancer therapy, and lower participation in clinical trials [34,72]. Conversely, individuals without any form of health insurance or with inadequate coverage face an increased likelihood of being diagnosed at advanced or metastatic stages and experiencing earlier mortality [16]. Alarmingly, one in four cancer patients with coverage in the United States expresses concerns about affording care, depleting savings to pay for treatment, and being unable to afford necessities such as housing, heat, or food due to cancer care costs [73]. These financial burdens, often correlated with higher-stage diagnoses, have proven to be a persistent driver of disparities.

Moreover, patients with public insurance frequently experience lower-quality care and less rigorous follow-up compared to those with private insurance, which is a critical determinant of overall mortality. Even after controlling for socioeconomic factors, differences in access to care and survival based on insurance type persist, as evidenced by a study of women with invasive breast cancer [74]. This study underscores that insurance type remains a significant factor even when income does not. An estimated 26% of the 12.5 million non-elderly adults who remain uninsured in the U.S. meet the standard criteria for Medicaid (https://www.kff.org/cc843f6/ (accessed on 17 March 2025)). Furthermore, the proportion of individuals enrolled in private insurance prior to diagnosis varies significantly by race for five of the six most common invasive cancer types in five states, indicating that insurance differences may exacerbate well-known disparities [75].

Numerous studies have elucidated differential access to treatments based on race and socioeconomic status, determined by insurance coverage, including participation in clinical trials. While United States policies have largely focused on extending insurance to improve cancer outcomes and research engagement [76], strategies such as eliminating co-pays, reducing trial costs, or addressing unrepresentative enrollment for only one subset of underserved patients will not fully tackle the root causes of health disparities. Comprehensive healthcare reform is essential to remove insurance type and its coverage-driven effects on out-of-pocket expenses and treatment delays, barriers that exclude large segments of the population from high-quality care and research [77]. Ultimately, this reform should ensure equitable access to cancer care for all, regardless of insurance status.

## 2. Systemic Barriers to Access

Access to quality cancer care is hindered by a spectrum of social, structural, and psychological challenges at individual, systemic, and policy levels. People living in rural or medically underserved areas face significant geographic challenges in accessing knowledgeable cancer specialists and state-of-the-art healthcare facilities [78]. Research indicates that Medicaid and uninsured patients in rural areas are the least likely to seek treatment at high-quality cancer centers [79]. Additionally, private insurance and place of residence are strong predictors of survival [80]. Beyond these physical barriers, many in underserved groups report feeling intimidated or shy when communicating with healthcare professionals [81]. Clinicians have also expressed discomfort discussing cancer genetics with patients of color [82]. Such implicit biases among healthcare providers can lead to subconscious judgments shaping patient interactions and treatment decisions [83]. These biases can influence behavior on both the employer and patient side, contributing further to inequities in healthcare access and outcomes. Health literacy is another critical factor. The effective use of diagnostic and treatment services requires individuals to read, comprehend, and analyze medical information, retrieve stored information, and draw informed conclusions. For patients with lower educational attainment or limited language skills, the complexities of cancer care can be overwhelming, leading to misunderstandings, misinformed decisions, and ultimately poorer health outcomes [84]. Efforts to reduce these barriers are multifaceted and include community-based programs, nationwide interventions in education and healthcare provision, and policy initiatives aimed at anti-discrimination [85]. Community-based programs can provide localized support and resources, helping individuals navigate the healthcare system and access necessary services. Nationwide educational interventions can improve health literacy, equipping individuals with the knowledge and skills needed to make informed health decisions. Policy initiatives are crucial for addressing systemic issues. Anti-discrimination policies can help ensure that all patients receive equitable care, regardless of their background or location [86]. Comprehensive healthcare reforms are needed to remove barriers related to insurance type and coverage, ensuring that all individuals have access to high-quality cancer care [87].

### 2.1. Geographic Disparities

Geographic disparities substantially impact access to cancer care services. Rural residents often have poorer access to cancer care compared to their urban counterparts due to fewer healthcare facilities and specialist availability [88]. Rural cancer care coordinators often manage greater workloads, and rural women must travel further for breast screening, leading to less frequent screenings [89]. Patients in rural areas are often diagnosed with more advanced diseases compared to urban patients [90]. Early diagnosis and access to better treatment can improve outcomes, making initiatives to facilitate earlier diagnosis and treatment in rural regions crucial. Rural patients also face increased transportation issues and financial burdens when traveling for cancer care [91]. Understanding how socioeconomic status, rural residence, and race/ethnicity intersect is essential for addressing these disparities. Telemedicine, patient navigators, and mobile services show promise in bridging these gaps and expanding access to quality cancer care in rural communities [92,93,94].

### 2.2. Healthcare Provider Bias

Systemic barriers in cancer care are influenced by both conscious and unconscious provider biases. Studies show that both conscious and unconscious biases affect clinical presentation and treatment [95]. Unconscious racial and socioeconomic biases can prompt delays in diagnosis, reduced trust, and avoidance of patient contact, directly impacting clinical outcomes. Meta-analyses reveal that implicit and explicit biases lead to prejudiced clinical decision-making, regardless of personal claims of tolerance [96]. Tests have documented pronounced negative biases against various minority groups, including women, LGBTQ individuals, immigrants, and disabled groups [55]. Since awareness training alone is insufficient to mitigate these biases, new strategies are needed to standardize patient treatment.

### 2.3. Lack of Health Literacy

Health literacy, the ability to read, understand, and act on medical information, is pivotal for effective cancer care. Low health literacy decreases one’s understanding of medical information and instructions, leading to more emergency room visits, longer hospital stays, and less use of preventive services [97]. Factors affecting health literacy include socioeconomic status, education, occupation, and language proficiency [98]. Certain racial and ethnic groups disproportionately experience low literacy rates, compounding barriers to cancer care [98]. Improving health literacy is critical for all racial and ethnic groups [99]. Community-driven and health education programs can help patients and their family engage more confidently with cancer care, leading to more informed decisions. Screening programs and educational initiatives are particularly valuable for improving cancer health literacy and boosting patient outcomes. Effectively addressing these overlapping barriers to quality cancer care demands a comprehensive approach that tackles social, structural, and psychological impediments [17]. Improving access to knowledgeable specialists, enhancing health literacy, and implementing equitable policies are crucial steps. By recognizing and addressing these barriers, we can ensure that every individual receives the quality cancer care they need and deserve.

## 3. Impact of Disparities on Cancer Outcomes

The first measurable evidence that inequalities in access to care lead to worse outcomes in cancer patients is the link between these disparities and delayed diagnosis [100]. Disadvantaged populations, those who are poorer and less likely to have private medical insurance in the U.S., are consistently diagnosed at more advanced stages of disease [33]. As overall cancer incidence decreases, the correlation between poverty and stage at diagnosis also tends to decline. Patients with more advanced cancers have worse outcomes due to the higher stage-related risk of death and the reduced effectiveness of available treatments for advanced tumors [101]. Income and medical insurance status are closely tied to the stage at which cancer is diagnosed, which in turn affects survival rates due to differences in the quality of cancer care [32]. Disparities in treatment include delays in referral to specialist services, the speed at which symptoms are investigated, the appropriateness of diagnostic tests and biopsy sites, and the receipt and effectiveness of adjuvant treatments [102]. These structural components of the cancer care pathway, along with demographic and socioeconomic factors, also influence access to other care interventions such as pain management and palliative care [103]. For example, patients with the same disease and trial measurements who experience reduced pain or improved performance status tend to have better survival outcomes [104]. Survival rates and disparities in survival are also linked to the origin of cancer, often reflecting differences in incidence. Disparities extend to all clinical outcomes in unselected populations of cancer patients, including unmeasured factors that impact therapy options, such as treatment preferences and toxicity [105]. Data consistently show that patients diagnosed at a more advanced stage have poorer clinical outcomes. An analysis of a cancer registry revealed that patients with regional-stage cancer at presentation had a 25% higher chance of dying from cancer compared to those with localized disease, even when controlling for treatment differences. Patients with advanced-stage cancer had a 127% higher chance of dying from cancer. Those diagnosed at an early stage not only had longer overall survival times compared to those with locally advanced and metastatic cancer but also longer cancer-specific survival [106].

### 3.1. Delayed Diagnosis and Advanced Stage at Presentation

In this section, we explore how various disparities intersect to result in delayed cancer diagnoses and more advanced stages at presentation. We examine the underlying reasons for these delays and highlight the significant impact on patient outcomes. Despite overall improvements in cancer diagnosis and mortality over the past century, disparities persist, particularly in the late presentation of the disease [107]. Ethnic disparities in cancer stage and patient outcomes can often be attributed to differences in access to healthcare [108]. One major factor contributing to these socioeconomic and access disparities is the delayed presentation and diagnosis of serious health issues. The later the diagnosis occurs in the course of disease, the worse the prognosis, as treatment becomes more challenging, outcomes are poorer, and survival rates decrease [109]. A lack of education and access to regular health services are key factors leading to delayed cancer diagnoses [12]. This delay can be linked to an uninsured attitude towards healthcare, where individuals may not seek medical attention promptly. Chronic illnesses often have subtle symptoms early on, which can be overlooked. Cancer diagnoses typically occur after a physical exam or diagnostic test reveals the disease. The initial step in obtaining a diagnosis is the patient’s interaction with the healthcare system, usually through a visit to a primary care provider [110]. The next step involves coverage of preventive care services through insurance. Reducing the number of patients presenting with advanced disease could significantly improve overall cancer survival statistics [111]. The stage and grade of the disease are the two best predictors of five-year survival and overall cure rates in most cancers [112]. The more advanced the stage and higher the grade, the harder it is to achieve a “cured” status. In clinical trials, where diseases are often identified earlier than in standard care, it is easier to determine if an investigational agent extends disease-free progression or survival. Additionally, lower-stage diseases allow for more techniques to minimize therapeutic side effects. For example, organ preservation is more successful in lower-stage diseases [113]. Immunotherapy combined with biologics and other curative treatments may be more effective when administered as primary therapy in patients with minimal cancer burden [114]. Even for patients with poor prognoses, those with lower disease burden tend to fare better than those with higher burden. Overall, addressing smaller challenges is more manageable. Many factors contribute to the stage at clinical presentation and grade of cancer. Reducing or eliminating unfavorable socioeconomic, racial, and insurance coverage variables requires a multifaceted approach at the healthcare systems policy level [115]. Denial of these issues is the first problem that needs to be addressed.

### 3.2. Differences in Treatment Options and Quality of Care

In a fee-for-service healthcare system, patterns of lower-quality care are prevalent among patients from lower socioeconomic strata, those who are underinsured or uninsured, racial and ethnic minorities, and individuals with lower educational levels [116]. These disparities manifest at various stages of the care process, although there is less data available for treatment regimens, particularly for childhood cancer and adult cancer medical procedures. Lower quality care is believed to negatively impact treatment adherence and outcomes. For instance, patients with inadequate support systems, whose ability to adhere to demanding treatment schedules is limited by work or childcare responsibilities, may avoid, discontinue, or delay more toxic regimens in favor of less intense outpatient treatments [117]. Terms like “patient preference” can sometimes obscure significant differences in reimbursement strategies, recruitment outreach, nursing support, monitoring, and options available to different groups, especially racial and ethnic minorities, the poor, the uninsured, the less educated, the low-income elderly, and those living in rural areas [118]. There are also disparities in the training and research focus of specialists and surgeons regarding these issues [119]. All cancer patients face challenges in receiving guideline-based care, but patients from marginalized communities experience a greater disparity, especially in access to advanced treatments [120]. Improving the quality of therapeutic cancer care requires addressing basic institutional inequities within the healthcare system. Enhancing access to treatment options is crucial for reducing disparities in the stage at diagnosis and, consequently, disparities in survival outcomes [121]. Narrowing these disparities by increasing access to effective care or addressing their root causes necessitates a fundamental shift in national policy direction and a deeper understanding of bias and discrimination within healthcare [122].

### 3.3. Survival Rates and Mortality Disparities

The differences in cancer survival rates and mortality among demographic subgroups are substantial. Five-year survival rates for all cancer sites are generally highest among non-Hispanic whites, while African Americans have the lowest five-year survival rates [123]. Age-adjusted statistics reveal that African Americans die at a disproportionately higher rate than the rest of the U.S. population [124]. This disparity spans various cancer types with differing mortality rates. For instance, the colorectal cancer mortality rate for African American men is 42% higher than that of Caucasian men [125]. These disparities result from longstanding policies related to healthcare, civil rights, and systemic racism, which influence numerous social, cultural, educational, and economic determinants [126]. Addressing these disparities requires shifting from an individualist paradigm in biological research, which views diseases as molecular aberrations within individuals, to a public health perspective that considers social determinants of health. While access to healthcare and timely treatment are crucial, they are not the sole contributors to disparities in outcomes [19]. Worse survival rates, even after accounting for treatment, are often linked to less appropriate and less aggressive treatments [15]. The increasing diversity of the U.S. population further exacerbates healthcare disparities, as each year’s survival rate builds on those who survived in prior years. Delayed diagnoses and later-stage detection significantly decrease survival outcomes. Increasing coverage and providing higher subsidies for doctors can improve access to screenings, diagnosis, and timely medical intervention [31]. Equitable access to clinical trials and community outreach programs tailored to individuals’ culture, age, and social determinants can improve survival rates across all forms of cancer. The COVID-19 pandemic has underscored that the health of the many impacts the well-being of the few [127]. Until everyone, regardless of gender and ethnicity, receives timely cancer diagnosis and treatment, our healthcare system remains as strong as its weakest link.

## 4. Barriers to Participation in Clinical Trials

Advancing cancer understanding and treatment hinges on clinical trials, but their full potential is hindered by an ongoing challenge: the slow and unrepresentative recruitment of participants, which directly limits the generalizability of findings to the broader population. This lack of diversity means that the benefits and risks observed in trials may not accurately reflect how new treatments will perform in all patients, potentially widening health disparities. Several factors contribute to this problem. Clinical trials are often inaccessible to individuals in rural areas, a common home for socioeconomically disadvantaged populations [128]. The initial requirement for consent before receiving study treatments, combined with the time and travel demands of ongoing appointments, creates a significant burden, especially where specialized treatments are not readily available. The limited awareness of clinical trial availability among both patients and some healthcare providers further restricts participation, particularly in these rural regions [128]. This issue is exacerbated by a lack of programs in rural areas designed to support low-income cancer patients in trial participation. The mistrust of the healthcare system presents another substantial barrier, particularly among communities who may perceive clinical trials as exploitative towards minority and vulnerable populations [129]. Interestingly, a study examining attitudes toward clinical trials found that White individuals reported a higher level of fear regarding potential side effects and exploitation compared to other groups. This challenges the common assumption that negative attitudes are primarily linked to ethnic and racial minorities, the elderly, or those from lower socioeconomic backgrounds [130]. To enhance the generalizability of clinical trial findings and ensure that all cancer patients can benefit from the latest research, addressing these barriers is crucial. Expanding and ensuring equitable access to clinical trials is a critical component of reducing disparities in cancer care. This necessitates efforts to raise awareness, implement supportive programs, and foster trust within diverse communities to encourage participation. By improving the inclusivity of clinical trials, we can develop more effective cancer treatments that truly serve all populations.

### 4.1. Underrepresentation of Minorities in Clinical Trials

Minorities remain significantly underrepresented in clinical trials, which limits the generalizability of research findings on the effectiveness, side effects, and quality of cancer treatments [130]. The adequate representation of racial and ethnic subgroups in clinical trials is essential to evaluate the safety and effectiveness of cancer treatments across different populations. However, the continued underrepresentation of minority populations poses a challenge in determining whether cancer treatments improve patient outcomes equally across various racial and socioeconomic subgroups [14]. This underrepresentation may also lead to underestimates of reported toxicity and poor treatment efficacy in minority groups. Several factors contribute to the underrepresentation of minorities in clinical trials. Some minority individuals may be unaware of clinical trials, while others may refuse to participate or be ineligible due to comorbidities [131]. Historical mistrust in clinical trials, stemming from unethical research practices, physical barriers to trial enrollment, lack of transportation, and the inability to take time off work are significant issues [132]. This problem has increasingly caught the attention of federal funding agencies and cancer care organizations, prompting several initiatives to improve diversity in clinical trials and increase the representation of minority and underserved patients. The underrepresentation of minorities in clinical trials adversely impacts study design and the recommendations generated [133]. For instance, an analysis of 168 U.S. phase 3 cancer trials revealed persistent disparities in racial and ethnic representation. Whites were overrepresented (+6.8%), while Blacks (−2.6%), Hispanics (−4.7%), and Asians (−4.7%) were underrepresented compared to cancer incidence rates. These findings highlight the need for improved minority inclusion to enhance trial equity and generalizability [134]. This lack of inclusiveness in cancer research highlights the need for regulators to ensure that studies more accurately reflect the unique cancer vulnerabilities of an increasingly diverse population [135]. Guidelines should be updated to ensure the frequency of underrepresented subpopulation data in clinical trials is transparent and detailed within subgroups [136]. Equitable recruitment of underrepresented patient subpopulations, including children, should be aggressively pursued. Coordinated approaches to enhance recruitment from and partnerships with health services, strengthening existing collaborations, and building new ones with Tribal Nations and regional urban jurisdictions are essential to improve access to healthcare resources and identify potential collaborative initiatives [137]. Leveraging network resources to create cross-network education and outreach activities can raise awareness about the significance of engaging in clinical research and the rewards and risks involved. By addressing these barriers, we can ensure that all cancer patients benefit from the latest research and treatments, ultimately improving survival outcomes across all populations.

### 4.2. Financial Barriers

Aside from the demands on an individual’s time and emotional health, substantial financial barriers frequently prevent individuals from participating in clinical trials [138]. This is not surprising, as most uninsured or underinsured individuals in the United States belong to lower-income families. The costs associated with participating in clinical trials can be considerable. Patients often need to travel long distances to participate, and some treatments received as part of clinical trials may not be reimbursed by insurance companies [139,140]. Lost wages are another major factor that may deter patients from participating. For those undergoing active cancer treatment, often the very individuals who could benefit most from access to new and potentially better treatments, clinical trial participation is simply out of reach. These financial barriers are not evenly distributed and reflect existing disparities. Removing these financial constraints would lead to greater participation from socioeconomic, racial, and ethnic minorities, as well as uninsured and underinsured individuals [141]. The same obstacles that prevent participation in clinical trials also likely deter individuals from seeking care altogether. Failing to address these obstacles grossly underrepresents these communities in the mainstream clinical trial system, impairing the generalizability of clinical trial results and, more insidiously, global rights to medical advancement. Many advocates for public health often overlook these issues. Policymakers, state governments, and national organizations should devote more resources to strategies that address the numerous barriers to cancer patient clinical trials [142]. Achieving true health equity in the United States requires overcoming these economic barriers. The constraints that drive people who could benefit from therapy to forgo an entire pathway of potential treatment options disproportionately affect already marginalized communities. Addressing financial obstacles in clinical trial research is essential for enabling broader access to cancer treatment and improving outcomes for all population.

### 4.3. Access to Information and Awareness

Globally, several issues contribute to the low enrollment rates in clinical trials, including limited awareness [143]. Low primary care provider (PCP)-to-patient ratios partly explain why non-academic practice oncology (PO) sites accrue fewer patients compared to academic practice oncology (APO) sites [144,145]. Patients with poor knowledge of clinical trials often have lower educational attainment and incomes [146]. Providing clear, accessible cancer information that meets international standards and expanding the pool of professionals involved in patient accrual are crucial steps. Those least likely to have information about clinical trials are also those most likely to receive lower-quality cancer treatment. It is misleading to blame patients alone for their exclusion from clinical trials, as they are often not offered the opportunity [147]. This is particularly true in minority and low-socioeconomic-status (SES) communities, where oncologists may propose fewer trials rather than patients refusing to participate. Effective communication about clinical trials can increase enrollment, while concerns related to trials can decrease it. Good communication is crucial in the eligibility process and can help mitigate concerns about clinical trials [13]. Medical jargon and complex eligibility criteria are substantial barriers [148,149]. Patients need information presented in patient-friendly language and time to absorb it. They also need assistance in understanding the meaning of terms and how they apply to the trial [150]. It is unfair to present people with documents they cannot understand, let alone translating complex documents into accessible language. Evidence suggests that integrating patient perspectives can help researchers develop new treatments and encourage broader participation. Investing in community engagement initiatives that increase flagged patient enrollment in clinical trials by even a small percentage is not enough if the fundamental issue is the perception of healthcare providers. Many cancer patients often describe their relationship with their healthcare providers as a partnership [151]. Engagement strategies need to be more nuanced. Donor staff may need essential cancer genetics sessions to increase confidence in genetics terminology before offering engagement opportunities [152]. When participants are given knowledgeable health practitioners, they can better understand and trust the information provided. Addressing these barriers requires a comprehensive approach that includes improving communication, providing patient-friendly information, and fostering trust between patients and healthcare providers. By doing so, we can increase clinical trial participation and ensure that all patients have access to the latest cancer treatments.

## 5. Strategies to Address Disparities

Identifying disparities in the literature is crucial, but finding potential solutions is even more important. These solutions should be multifaceted, addressing immediate concerns like financial barriers to accessing care, as well as root causes such as racism [153]. Effective programs often focus on community-specific needs, as there is no one-size-fits-all approach. Many successful programs employ community health workers who act as liaisons between patients and the healthcare system, providing education, support, and advocacy [154]. These workers often come from the communities they serve, which helps build trust and improve communication. Additionally, providing social support services, such as transportation assistance, childcare, and financial counseling, can help address some of the practical barriers that prevent individuals from accessing care [155]. Healthcare providers must also address aspects of their own behavior that contribute to disparities. This includes recognizing and mitigating implicit biases, refining communication skills, and creating a more inclusive and respectful environment for all patients [156]. Training in cultural competency is essential, as it helps providers understand and respect the cultural backgrounds of their patients, leading to more effective and personalized care. System-wide changes can significantly reduce disparities [30]. Improving education in underserved populations is a key objective. This can involve delivering health education in schools, community centers, and through public health campaigns. Improving access to care is another critical area. This might include expanding Medicaid, increasing the number of healthcare providers in underserved areas, and ensuring that all patients have access to preventive services and early detection programs. Several successful policy interventions have been reported, demonstrating the effectiveness of these approaches. For example, policies that provide funding for community health centers, support for telemedicine, and incentives for healthcare providers to work in underserved areas can help reduce disparities [157]. Additionally, policies that address social determinants of health, such as housing, education, and employment, can have a profound impact on health outcomes [158]. Engaging community organizations is a critically important tool in any disparities program. Progress in social change in the United States has often been achieved through the joint efforts of professionals in government, non-profit organizations, and grassroots movements [159]. These groups may not initially share the same goals, but their collaboration over time has led to profound impacts on vital issues, benefiting society. Community organizations can provide valuable insights into the specific needs and challenges of the populations they serve, and they can help design and implement effective interventions. By implementing these strategies, we can work towards reducing disparities in cancer care and ensuring that all patients receive the quality care they deserve. This requires a concerted effort from all stakeholders, including healthcare providers, policymakers, community organizations, and patients themselves. Together, we can create a more equitable healthcare system that meets the needs of all individuals, regardless of their background or circumstances [160].

### 5.1. Community-Based Interventions

There are many strategies for addressing disparities in access to cancer care, and a key part of the solution likely resides outside the traditional healthcare system. Community-based interventions, developed, implemented, and sustained by local members, are designed to be culturally, linguistically, and resource-appropriate for the populations they serve [161]. Several such programs have successfully increased access to cancer treatment. A substantial body of data documents numerous programs that have increased cancer screening uptake among various underserved groups [162]. This is crucial because screening is the first step in diagnosing and treating cancer. These successful programs demonstrate that local healthcare providers, public health agencies, and community institutions are eager to address cancer disparities [163]. They can serve as models for designing systems that bring patients more effectively to cancer diagnosis and treatment centers. Community-based interventions are also valuable from the perspective of interviews with patients, cancer survivors, and individuals who have interacted with the healthcare system while seeking care for family members or acting as “cancer navigators” [164]. These interviews can highlight other, previously unidentified barriers to accessing the healthcare system. Focusing on this part of the healthcare system has proven effective in raising awareness and increasing patient use of healthcare services after they become aware of symptoms and seek initial workup and testing for possible cancer [165]. Engaging local individuals facing cancer, regardless of where they are on their cancer journey and with whatever resources they have, has great potential to effect meaningful and needed change [166]. Successful community-based programs have shown the importance of working closely with local clinicians and healthcare organizations to disseminate information and offer access to services [167]. They also emphasize the need to develop mechanisms and protocols to ensure continuity of care between community outreach and healthcare delivery or research components [168]. For example, successful partnerships often include clinical care, emotional, financial, and social support networks for individuals navigating the healthcare system [169]. Programs in small serving area collaborations need to plan effective strategies for pooling and leveraging resources. A key research question is how to evaluate the efficacy of community-level interventions that address multiple factors using relevant content, process, and structural measures [170]. Experience with such programs shows that successful interventions are generally developed through the active engagement of stakeholders who possess local support and make key decisions about needed changes and resource allocation. These stakeholders are directly affected by the proposed intervention through its design and outcomes. Once clinical, research, and other field-based programs are operational, interactions with the medical community will likely fine-tune the intervention, possibly triggering additional assessment or initiative development research. Evaluating the tipping points for minority and underserved cancer care disparity research involves discussing all recommended efforts. By implementing these strategies, we can work towards reducing disparities in cancer care and ensuring that all patients receive the quality care they deserve.

### 5.2. Cultural Competency Training for Healthcare Providers

Incorporating cultural competency training for healthcare providers is crucial for reducing disparities in cancer care [171]. Cultural competency is the ability to understand, honor, and respect the beliefs, lifestyle, values, and needs of patients from different races, cultures, or religions. Implicit biases—unconscious beliefs about someone’s race, ethnicity, age, gender, appearance, language, or sexual orientation—can negatively impact the healthcare provider–patient relationship [68]. Cultural competency training helps healthcare providers become aware of these biases, enabling them to reduce their own implicit biases and the resulting inequities. Studies have shown that cultural competency training improves patient–provider interactions in healthcare settings [172]. For example, interpreters who received cultural competency training were significantly more likely to build rapport with their clients. This demonstrates that even brief cultural competency training can have a meaningful impact on individual patient–provider interactions. Cultural competency involves not only knowledge about others but also self-awareness, an understanding of healthcare, and societal context. By increasing the awareness of biases, healthcare providers can minimize these biases, leading to benefits for patients such as increased trust in the provider, better treatment adherence, greater satisfaction with care, and higher patient retention [173]. To increase the cultural competency of healthcare professionals, education should begin in medical school and continue through training [174]. Postgraduate training may include specialized programs in certain regions and interdisciplinary programs involving both healthcare and public health training. In medical and counseling professions, cultural competency is often referred to as cultural competence, cultural diversity, and culturally competent care [175]. There are several examples of cultural competency training for healthcare providers in the literature. One of the most comprehensive cultural competency training programs in the United States teaches healthcare professionals how to communicate effectively with diverse patient populations. By implementing cultural competency training, healthcare providers can better serve diverse populations, ultimately reducing disparities in cancer care and improving outcomes for all patients [176].

## 6. Policy Recommendations

**Figure 1:**
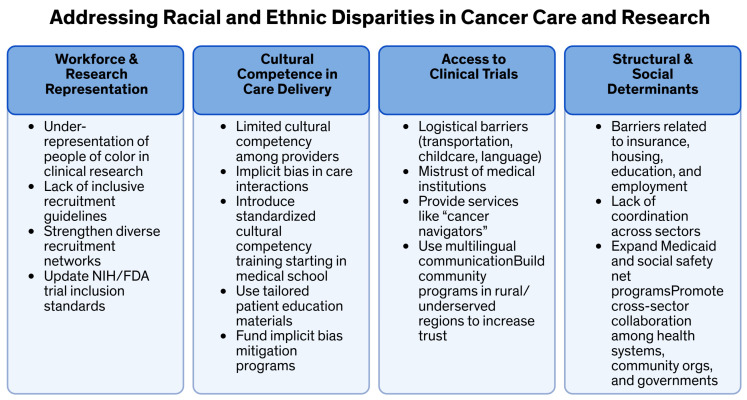
Addressing racial and ethnic disparities in cancer care and research. This figure summarizes major barriers to equitable cancer care and clinical trial participation across four domains, along with actionable solutions informed by the current literature.

**Figure 2:**
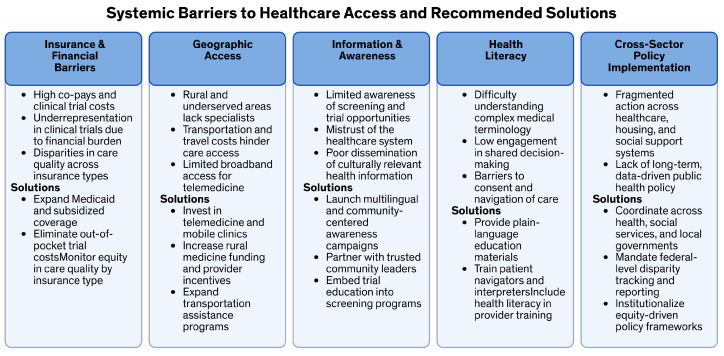
Systemic barriers to healthcare access and recommended solutions. This figure summarizes major systemic barriers to equitable healthcare and clinical trial access, with proposed strategies to address them.

To address socioeconomic, racial, and insurance-based disparities in cancer care, systemic societal-level policies, not just practice and medical care guidelines, need to be implemented. These policies should be institutionalized and long-term. First, increasing access to care is crucial [177]. This can be achieved by expanding state Medicaid programs, increasing funding for community-based programs, and enhancing poverty subsidizations from local to national levels [69,70]. These measures will help ensure that more individuals have access to the necessary healthcare services. Second, systematic data collection is essential. Data should be collected and analyzed at the federal, community, and hospital levels and then presented to lawmakers to inform policy decisions [178]. This will ensure that governmental actions are based on accurate and comprehensive information, ultimately providing universal access to cancer care and clinical trials. Third, collaborative guidelines should be established. Federal and healthcare training departments should create guidelines that promote collaboration among multiple stakeholders, including healthcare providers, service organizations, community organizations, and governmental agencies. Engaging in stakeholder education campaigns will help reduce barriers to cancer care, prevention, and diagnosis [179]. Fourth, increasing diversity in clinical trials is vital. Mandating increased racial and socioeconomic diversity among federal clinical trial participants is necessary. Additionally, fostering university partnerships, continuing education, community hospital partnerships, and regional multidisciplinary case discussions will ensure consistent, standardized cancer care nationwide. Including underserved populations in cancer research will provide access to the latest anti-cancer agents [180,181]. Finally, bench-to-bedside funding should be developed. Federal and other grant-giving bodies should create funding that spans from pre-clinical ethical research to long-term community-based surveillance studies. This funding will support continuous systematic cancer care research that pushes cancer cure rates higher [182]. Implementing these systemic societal-level changes could significantly reduce disparities in cancer care [18]. Future practical and research efforts should continue to explore ways to combine practical applications and research to achieve true health equity [67]. By following these recommendations, we move towards a more equitable healthcare system that ensures all patients receive the high-quality care they deserve (Table 3, Figure 1 and Figure 2). These recommendations are primarily relevant to the USA and are a specific limitation of this study.

## 7. Future Directions and Political Will

**Figure 3:**
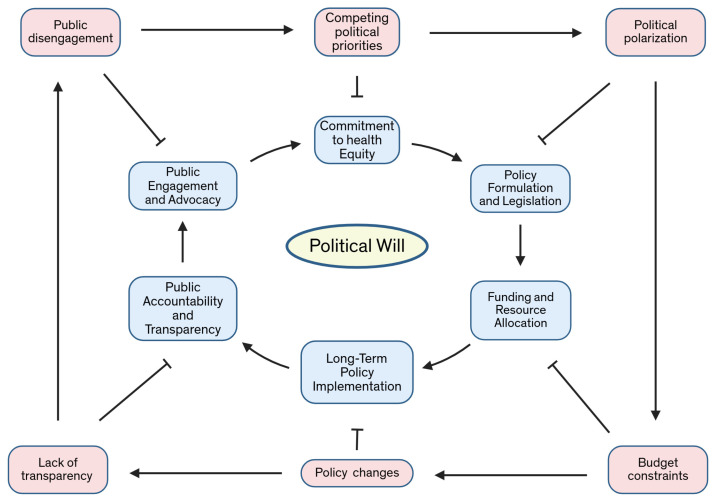
Building political will: A framework for action against healthcare disparities. This figure presents a framework illustrating the steps required to cultivate political will, from raising awareness and building consensus to enacting policies and ensuring accountability, while highlighting the potential inhibitory factors at each stage. Note: This conceptual framework was developed by the authors through a thematic synthesis of the health equity and policy literature. It highlights the necessary components to build political will and associated barriers [25,96,102].

The combination of the Great Recession, the substantial expansion of insurance coverage, and newly legalized patient assistance program advertising may lead to significant improvements in access to cancer diagnosis and care for economically vulnerable individuals. This development has the potential to revolutionize the healthcare industry and improve the lives of millions. However, for these advancements to have a lasting impact, it is essential to address not only economic and health policies but also commit to increasing the diversity within the cancer workforce and enhancing the access to logistical support programs [190,191]. By prioritizing innovative therapies and acknowledging market failures that prospective trial participants may not recognize, we can ensure equal access to cutting-edge treatments and breakthroughs. It is also crucial to remain vigilant about surges in cancer diagnoses that might result from disability policies or environmental disasters due to an insufficient political will. Identifying and addressing these underlying causes allows us to take proactive measures to minimize their impact and support those affected. To meaningfully reduce racial disparities in cancer burdens and promote health equity, our efforts must focus on early detection and prevention of select cancers. This involves implementing comprehensive screening programs targeting at-risk populations and providing the necessary resources for timely and accurate diagnoses [187]. Additionally, reducing preventable cancer risks through education, awareness campaigns, and policy changes is paramount [185]. By adopting this multifaceted approach, we can create a society where cancer does not discriminate based on socioeconomic status or race (Table 4). We have the tools, knowledge, and resources to make significant strides in this area, but broad collaboration from all sectors is necessary. Working together, we can envision a future in which cancer is no longer disproportionately borne by the economically vulnerable, but rather a disease substantially minimized through compassion, innovation, and collective action [192] (Figure 3).

## 8. Conclusions

In conclusion, addressing disparities in cancer care requires a comprehensive and multifaceted approach that encompasses economic, social, and systemic changes [2]. Expanding access to care through Medicaid and community-based programs, improving cultural competency among healthcare providers, and enhancing diversity in clinical trials are essential steps [189,193,194]. Systematic data collection and collaborative guidelines can guide policy decisions and promote equitable healthcare practices [186]. Community-driven interventions tailored to the specific needs of underserved populations have shown success in increasing cancer screening and treatment uptake [184]. Additionally, addressing financial barriers and improving logistical support can ensure that economically vulnerable individuals have access to cutting-edge treatments [188]. By focusing on early detection, prevention, and education, we can reduce avoidable cancer risks and promote health equity. Achieving these goals necessitates coordinated efforts from all sectors of society, leveraging existing tools, knowledge, and resources to create a future where cancer care is accessible and equitable for all [183]. Through compassion, innovation, and collective action, we can minimize the burden of cancer and ensure that no one is disproportionately affected by this disease.

## Figures and Tables

**Table 1:** Inclusion and exclusion criteria for literature selection.

Criteria Category	Inclusion Criteria	Exclusion Criteria
Population	Studies focusing on human populations affected by cancer, particularly those addressing health disparities in marginalized or underserved groups.	Studies not directly related to human cancer or cancer care.
Intervention/Exposure	Research examining socioeconomic status, race/ethnicity, and/or insurance status as primary factors influencing cancer care access, outcomes, or disparities.	Studies that do not primarily investigate socioeconomic status, race/ethnicity, or insurance status as factors influencing disparities.
Outcomes	Studies reporting on cancer incidence, prevalence, morbidity, mortality, survivorship, quality of life after cancer treatment, screening rates, stage at diagnosis, access to medical resources, or clinical trial participation.	Studies with irrelevant outcomes or those not focused on disparities.
Study Design	Original research articles (quantitative, qualitative, mixed-methods), systematic reviews, meta-analyses, and relevant policy analyses or reports from recognized health organizations.	Non-empirical articles such as opinion pieces, editorials, commentaries, or conference abstracts (unless specifically sought as the gray literature).
Language	English-language publications.	Non-English-language publications.
Publication Date	Published between January 2000 and December 2023. Some relevant studies outside this range were selected based on impact.	Publications outside the specified date range.

**Table 2:** Cancer disparities across racial and ethnic Groups: This table outlines observed disparities, contributing social and healthcare-related factors, and actionable strategies to reduce inequities in cancer outcomes.

Category	Key Insights
Observed Disparities	Black men: Highest rates Native Hawaiians: Higher rates, later diagnosis Asians: Specific cancers like liver and stomach
Social Determinants	Lower income, education, and healthcare access in minority communities
Healthcare Barriers	More uninsured/underinsured, fewer facilities, implicit bias in care
Equity-promoting Strategies	Recruit diverse participants in research, train providers in cultural competence, policy reforms [14,26,59,68]
Policy and Practice Recommendations	Improve care access, increase research participation, address social determinants [19,27,30,69,70]

**Table 3:** Disparities in cancer care and clinical trials: This table illustrates the multifaceted disparities affecting cancer care and clinical trial participation, categorized into racial and ethnic disparities and systemic barriers to access.

Category	Specific Barriers	Type of Barrier	Policy and Intervention Recommendations
Cultural Competence and Training	Lack of culturally competent healthcare professionals. Insufficient training on diverse patient needs. Mistrust due to historical injustices [81,107,129,140].	Cultural/Educational	Mandate cultural competency training in medical education and healthcare institutions [60,146,173,176]. Increase diversity in the healthcare workforce [18,30,138,153]. Develop community-based programs to build trust [161,164].
Policy and Systemic Factors	Social determinants of health (housing, education, employment). Lack of data-driven policy decisions. Insufficient funding for community programs [183,184,185].	Systemic/Policy	Implement policies addressing social determinants of health [18,19,30,160]. Enhance data collection on disparities to inform policy [145,186]. Increase funding for community-based cancer care and prevention [167,182]. Encourage collaboration between healthcare providers, community organizations, service agencies, and government bodies to shape future healthcare policies and engage in public education campaigns [179,187].
Insurance and Financial Access	Limited Medicaid coverage. High out-of-pocket costs for care and clinical trials. Financial barriers to accessing non-medical needs (transportation, childcare) [139,149,169,188].	Financial/Economic	Expand Medicaid eligibility and coverage [31,69,72,74,75,177,189]. Eliminate out-of-pocket costs for cancer care and clinical trials. Provide financial assistance for non-medical needs [177].
Geographic and Access Barriers	Underserved rural areas. Limited access to specialists and clinical trials. Transportation difficulties. Lack of telemedicine services.	Geographic/Access	Expand telemedicine services, especially in rural areas [94]. Increase funding for transportation assistance programs [91]. Improve access to specialists in underserved communities [94,187].
Health Literacy and Communication	Difficulty understanding medical information. Lack of patient-friendly communication. Implicit bias in healthcare interactions [96].	Interpersonal/Communication	Enhance health literacy programs [17,98,99,146,152]. Provide clear, accessible patient education materials [150,164]. Implement implicit bias training for healthcare providers [18,55,95,96]. Increase patient navigator support [93,117,169].

**Table 4:** Aspects of political will in addressing healthcare disparities: This table outlines key aspects of political will necessary to effectively address healthcare disparities, detailing the description, challenges, and recommendations for each aspect.

Aspect of Political Will	Description	Challenges	Recommendations
Commitment to Health Equity	Political leaders need to prioritize health equity as a central policy objective.	Competing political priorities, lack of sustained focus on marginalized communities.	Advocating for policy agendas that focus on reducing disparities and securing long-term legislative support [166,189,190,192].
Policy Formulation and Legislation	The creation of laws and regulations aimed at expanding access to healthcare, reducing disparities, and promoting inclusion in clinical trials.	Political polarization, insufficient stakeholder engagement, resistance from special interest groups.	Engage cross-party support and ensure stakeholder representation in policy design and implementation [20].
Funding and Resource Allocation	Government funding must be allocated to health programs targeting underserved populations and addressing disparities.	Budget constraints, political reluctance to increase healthcare spending, economic downturns.	Secure bipartisan agreements to prioritize funding for public health, Medicaid expansion, and research inclusion programs [22,69,70].
Long-Term Policy Implementation	Policies should be sustained and institutionalized to ensure ongoing support for healthcare equity.	Policy shifts with changes in administration, lack of enforcement of existing regulations.	Develop bipartisan, long-term strategies for health equity, enforce existing legislation, and ensure continuity across government terms [23,27,30].
Public Accountability and Transparency	Political leaders must be held accountable for the success or failure of health equity initiatives through transparent reporting and data-driven evaluation.	Lack of transparency in data reporting, limited mechanisms for accountability.	Establish independent oversight bodies and require regular reporting on healthcare access, clinical trial inclusion, and disparity reduction efforts.
Public Engagement and Advocacy	Political will can be strengthened by engaging the public and raising awareness about healthcare inequities.	Public disengagement, misinformation, and apathy toward healthcare reforms.	Launch public awareness campaigns, facilitate community engagement in policy discussions, and build coalitions to support health equity initiatives [2,18,24,96,143,173,176,180,181,183].
